# Comparative Genomics of the Mating-Type Loci of the Mushroom *Flammulina velutipes* Reveals Widespread Synteny and Recent Inversions

**DOI:** 10.1371/journal.pone.0022249

**Published:** 2011-07-20

**Authors:** Arend F. van Peer, Soon-Young Park, Pyung-Gyun Shin, Kab-Yeul Jang, Young-Bok Yoo, Young-Jin Park, Byoung-Moo Lee, Gi-Ho Sung, Timothy Y. James, Won-Sik Kong

**Affiliations:** 1 Mushroom Research Division, National Institute of Horticultural and Herbal Science, Rural Development Administration, Suwon, Republic of Korea; 2 National Academy of Agricultural Science, Rural Development Administration, Suwon, Republic of Korea; 3 Department of Ecology and Evolutionary Biology, University of Michigan, Ann Arbor, Michigan, United States of America; University of Minnesota, United States of America

## Abstract

**Background:**

Mating-type loci of mushroom fungi contain master regulatory genes that control recognition between compatible nuclei, maintenance of compatible nuclei as heterokaryons, and fruiting body development. Regions near mating-type loci in fungi often show adapted recombination, facilitating the generation of novel mating types and reducing the production of self-compatible mating types. Compared to other fungi, mushroom fungi have complex mating-type systems, showing both loci with redundant function (subloci) and subloci with many alleles. The genomic organization of mating-type loci has been solved in very few mushroom species, which complicates proper interpretation of mating-type evolution and use of those genes in breeding programs.

**Methodology/Principal Findings:**

We report a complete genetic structure of the mating-type loci from the tetrapolar, edible mushroom *Flammulina velutipes* mating type *A*3*B*3. Two mat*B*3 subloci, mat*B*3a that contains a unique pheromone and mat*B*3b, were mapped 177 Kb apart on scaffold 1. The mat*A* locus of *F. velutipes* contains three homeodomain genes distributed over 73 Kb distant mat*A*3a and mat*A*3b subloci. The conserved mat*A* region in Agaricales approaches 350 Kb and contains conserved recombination hotspots showing major rearrangements in *F. velutipes* and *Schizophyllum commune*. Important evolutionary differences were indicated; separation of the mat*A* subloci in *F. velutipes* was diverged from the *Coprinopsis cinerea* arrangement via two large inversions whereas separation in *S. commune* emerged through transposition of gene clusters.

**Conclusions/Significance:**

In our study we determined that the Agaricales have very large scale synteny at mat*A* (∼350 Kb) and that this synteny is maintained even when parts of this region are separated through chromosomal rearrangements. Four conserved recombination hotspots allow reshuffling of large fragments of this region. Next to this, it was revealed that large distance subloci can exist in mat*B* as well. Finally, the genes that were linked to specific mating types will serve as molecular markers in breeding.

## Introduction

The genes that regulate mating in fungi encode sets of regulatory and signaling molecules that are broadly distributed in eukaryotes. The corresponding pathways, which are often linked to pathogenicity and fruiting body formation, comparably control developmental processes in other organisms, such as pattern formation in development and sexual differentiation in animals. Studies in Agaricomycetes, that include many important mushroom-forming fungi, therefore also inform our collective understanding of cellular development in eukaryotes. Mushroom forming fungi are further important sources for food (mushrooms, fermentation), medicine (pathogens, fibers, health-promoting, anti-cancer products) and green technologies (waste recycling, fertilizers, bioremediation). As such they represent a massive economical asset and better understanding of their sexual propagation is desirable.

Mating is the beginning step in sexual development of mushroom-forming fungi. Their life cycle is characterized by haploid as well as diploid stages. Sexual, haploid spores that are dispersed by a mushroom develop into monokaryotic mycelia. Upon fusion of two genetically different monokaryons a dikaryotic mycelium is established and the different nuclei of the two mating partners coexist within the cells of the dikaryon during vegetative growth and production of fruiting bodies. Only upon maturation of the mushroom, the different nuclei fuse in specialized reproductive cells termed basidia and new haploid spores with separate mating-types are created (for a review see [1]). The heterothallic fungi restrict their self-mating by use of one (bipolar species) or two (tetrapolar species) incompatibility loci. This system supports out-breeding and helps to promote genetic variability in populations.

Our current knowledge on the molecular genetics of mating in mushrooms is primarily based on studies in the model organisms *Schizophyllum commune* and *Coprinopsis cinerea* ( = *Coprinus cinereus*). The mating-type loci in those species are termed *A* and *B* and control different developmental pathways that are required to maintain a fertile dikaryon (for reviews see [2], [3]). Each mating-type locus consists of tightly linked subloci and encodes multiallelic genes [4], [5], [6]. These alleles are highly polymorphic at DNA and amino acid (AA) levels [7], [8], [9] due to balancing selection that favors mating-type alleles that become rare and thus extends the coalescence time between alleles [10]. The *A* and *B* subloci are functionally redundant and heterozygosity at a single sublocus is sufficient to activate the respective *A* or *B* pathway.

*A* mating-type loci encode pairs of divergently transcribed homeodomain genes (HD genes) and are typically accompanied by the *Mitochondrial Intermediate Peptidase* gene (*MIP*) and a *Beta-flanking gene* [11], [12]. HD proteins are distinguished based on conserved DNA binding motifs; homeodomain 1 (HD1) and homeodomain 2 (HD2). HD1 proteins further contain two nuclear localization signals, an activation domain and only weakly bind DNA [13], [14] whereas HD2 proteins lack these domains but have strong DNA binding properties [15], [16]. Both HD1 and HD2 proteins possess N-terminal dimerization motifs that facilitate their interaction. Interaction of HD1 with a compatible HD2 protein generates a heterodimer that serves as a transcription factor for the *A* pathway [15], [17].

The *B* mating-type loci are comprised of pheromone receptors and pheromones (recently reviewed in [3]) and each pheromone receptor is accompanied by one or several pheromones in a sublocus [9], [18]. Pheromone genes encode small precursor proteins with C-terminal CAAX motifs (C = cysteine, A = aliphatic and X is any residue) that are farnesylated. They usually contain an acidic AA pair as well (ER or EH in *C. cinerea*) about 10–15 AA from the C-terminus. Those acidic residues are highly conserved in *C. cinerea* and have been speculated to be the site of proteolytic cleavage [5], [19], [20], [21], [22]. They are also conserved in other basidiomycete pheromones, though not in all [23], [24]. After farnesylation and proteolytic splicing, fungal pheromones typically constitute about 9–11 amino acid peptides [19], [25].

Fungal pheromone receptors are classified within the Rhodopsin-like superfamily and typically contain seven membrane spanning regions (7-TM). They are further characterized by a short N-terminal extracellular domain and a long cytoplasmic C-terminal tail [26]. The cytoplasmic domains are presumed to dock trimeric G-proteins that can activate the downstream *B* pathway after phosphorylation [26]. Phosphorylation of G-proteins is triggered by interaction of specific pheromones with extracellular pheromone receptor domains.

The fundamental composition of the *A* and *B* mating-type loci was found to be strongly conserved within basidiomycetes. The redundant subloci are a result of doubling during evolution and tetrapolar mating systems were found in all three major linages, suggesting an ancient origin [27], [28]. Moreover, bipolar and tetrapolar species have been shown to contain essentially the same genes [24], [29]. On the other hand, the new availability of genome sequences reveals significant variability within mating-type loci of basidiomycetes and their numbers of pheromone receptor, pheromone and homeodomain encoding genes differ greatly between species [12], [30], [31], [32]. To this, a new mating-type system that is not strictly bipolar or tetrapolar was discovered [30].

*Flammulina velutipes*, also known as Winter Mushroom and Enokitake is one of the major cultivated mushrooms in Asia. Beyond having a tetrapolar mating system with multiple alleles, the genetics of its mating-type system have remained unknown. We decided to elucidate the genetic structure of this important mushroom to map the mating-type genes and use comparative genomics to understand evolutionary relevant distinctions of the *F. velutipes* loci, as well as to implement this knowledge in our mushroom breeding programs. We obtained a complete map of the mating-type genes from *F. velutipes* KACC42780, identified the specific mat*A* and mat*B* loci and explain some of the events that caused the significant deviation of the mat*A* region in comparison to the model species. Mating-type defining genes are currently used to construct haploid, monokaryotic, mushroom producing strains and primers for PCR based mating-type identification.

## Materials and Methods

### Strains and culture conditions

*Flammulina velutipes* strains were cultivated at 25°C on 100×20 mm dishes (SPL, South Korea) containing Potato Dextrose Agar (HIMEDIA Laboratories, India) for two weeks. Stocks were transferred to fresh PDA dishes every three months, sealed (Clean Wrap) and stored at 4°C after three to five days growth (storage up to one year). For genomic DNA isolation, PDA was covered with cellophane (general household) prior to inoculation. For mating experiments, small dishes (60×15 mm, SPL) were inoculated with agar plugs (5×5 mm) at 0.5 cm from the center and grown for one week. Fresh plugs from the center of the plate that contained merged mycelia were subcultured for another four to eight days prior to clamp formation analysis. Mushroom cultivation in small bottle cultures was started with mating of two monokaryons as described above. Confirmed dikaryons were grown on a 30 mm layer of sterilized sawdust (Douglas Fir)/wheat bran (Rice) mixture (4∶1) in 25×45 mm glass bottles (three mycelial plugs/bottle). After 10 days incubation at 25° the surface of colonized wood was scraped off and bottles were filled with cold distilled water for 1 hr. Following upside down drying for 30 min bottles were incubated at 15°C, 95% humidity for three weeks. Spores were collected by placing the caps of the mushrooms in small dishes for one day and eluted in sterilized water prior to plating on PDA. Strains used in this study were KACC42780 (*A*3*B*3), KACC43777 (*A*4*B*4) and dikaryon KACC43778 (*A*3*B*3*A*4*B*4) available under Korean Agricultural Culture Collection (KACC), RDA, Korea. Monokaryons for segregation studies were all derived from dikaryon KACC43778. For analysis of mat*B* genes in *F. velutipes* strains from different geographical locations we used laboratory stock strains 4004-32 & 4004-23 (Korea); 4031-04 & 4031-10 (Korea, commercial strain Paengi-2); 4028-34 & 4028-38 (Taiwan); 4015-19 & 4015-11 (Japan); 4017-06 & 4017-05 (Korea); 4006-04 & 4006-01 (Korea); and 4023-01, 4023-05, 4023-29 & 4023-32, *F. velutipes* var. *longispora* (USA).

### Genomic DNA extraction

For isolation of genomic DNA, 400 µl of extraction buffer (100 mM NaCl, 50 mM EDTA, 0.25 M Tris-HCl, 5% SDS), 400 µl of 2x CTAB buffer (2% CTAB, 100 mM Tris-Hcl pH 8.0, 20 mM EDTA pH 8.0, 1.4 M NaCl, 1% polyvinyl pyrrolidone) and 500 µl phenol-chloroform-isoamylalcohol (25∶24∶1, Bioneer, South Korea) were added to 0.1–0.5 g of lyophilized or fresh mycelium and briefly vortexed. After 5 min incubation at room temperature (RT) samples were centrifuged at 13.000 rpm, 4°C for 5 min. The supernatant was mixed with 0.7 volumes isopropanol and centrifuged for 10 min, 4°C. After washing with 70% EtOH, air dried samples were eluted in 50–100 µl TE and treated with RNase A (Bio Basic Inc, Canada) for 30 min at 60°C.

### Southern hybridization

DNA probes (500bp) were amplified by PCR (primers; Table S2). Genomic DNA was digested to completion with *Sac*I at 37°C, over night. Agarose gels (0.8%) containing the digested gDNA were soaked in 0.25 M HCl for 20 min for depurination, 30 min in Denaturation buffer (0.5 M NaOH, 1.5 M NaCl pH 7.5) and 10 min in Neutralization buffer (0.5 M Tris–HCl pH 7.5, 1.5 M NaCl). DNA was transferred to a nylon membrane (Amersham) via capillary transfer with 10x SSC (1.5 M NaCl, 0.15 M sodium citrate) for 20 hrs and membranes were baked at 80°C [33]. Pre-hybridization and hybridization were done in a hybridization solution (5x SSC, 50% formamide, 5x Denhardt's solution, 0.5% sodiumdodecylsulfate and 25 mg/ml denatured salmon sperm DNA). Pre-hybridization was performed for 3 hrs and hybridization was continued for 24 hrs with a probe labeled with [a-32P] dCTP (Ladderman TM Labeling kit, Takara, Japan) at 42°C. The membrane was exposed to an imaging screen (Fuji) for 18 hrs and DNA bands were visualized using a personal molecular imager system (Bio-Rad, USA).

### PCR analyses and sequencing

PCRs were performed using GoTaq flexi kit (Promega Korea Ltd, South Korea) with specific primers (Bioneer, South Korea) for each gene (Table S2). Thermal cycling parameters consisted of 10 min denaturation at 94°C, 30 cycles of 94°C, 30 s denaturation, 30 s annealing (temperature 2°C lower than the specific, lowest melting temperature of primers used), 30 s extension at 72°C and a 10 min final extension at 72°C. Samples for sequencing were amplified with a BigDye Terminator cycle sequencing kit (Applied Biosystems, USA), precipitated with 0.1 volume 3 M Sodium Acetate (pH 5.8) and 2.5 volumes 100% EtOH for 10 min at −70°C and centrifuged for 15 min, 13000rpm at 4°C. Pellets were washed with 70% EtOH, air dried and eluted in Hi-Di Formamide (Applied Biosystems, USA) and analyzed on a ABI3730 DNA Analyzer.

### Identification of pheromone receptors, pheromone precursors and *CLA4*

A draft genome sequence of *Flammulina velutipes* monokaryotic strain KACC42780 was screened for the presence of pheromone receptors by tblastx searches with the sequence of pheromone receptor gene *Bar1* of *Schizophyllum commune* (National Center for Biotechnology Information (NCBI) Genbank accession number X77949.1), *Rcb1* of *Pholiota nameko* (AB201119.1) and *Rcb3* of *Coprinus cinereus* (CAA71962). The genome sequence of *F. velutipes* will become available at the Agricultural Genome information Center, RDA (http://10.30.100.11:8000/) though sequence downloads and search options may remain restricted. Gene models for pheromone receptor genes were manually annotated using tblastx derived homologues from NCBI. Secondary protein structures of *FvSTE3* encoded proteins were predicted using HMMTOP version 2.0 (Hungarian Academy of Sciences, http://www.enzim.hu/hmmtop/, [34] and TMHMM Server v.2.0 (CBS, Denmark, http://www.cbs.dtu.dk/services/TMHMM-2.0/, [35]. All *FvSTE3* containing contigs were fully screened for pheromone precursors. Open reading frames between 40 and 100 amino acids were manually analyzed for C-terminal CAAX motifs and screened using Pfam 24.0, http://pfam.janelia.org/, [36]. *CLA4* was identified in *F. velutipes* using *CLA4* of *Pleurotus djamor* (Genbank AY462110). NCBI Genbank accession numbers for identified genes of *F. velutipes* are: *FvSTE3.1* = HQ630590; *FvSTE3.2*/*FvPP2*/*FvPP3* = HQ630591; *FvSTE3.s1* = HQ630592; *FvSTE3.s2* = HQ630593; *FvSTE3.s3* = HQ630594; *FvSTE3.s4* = HQ630595; *FvSTE3.s5* = HQ630596; *FvPP1* = HQ630597.

### Identification of homeodomain proteins

The genome database of *F. velutipes* (see above) was screened for the presence of HD1 and HD2 proteins by tblastn searches with the sequences of *C. cinereus 5B6_b1-4* (NCBI Genbank AF126786.1), *C. cinereus* (JV6) *A42_b2-1* (NCBI Genbank X79687.1), *C. cinereus a42_d1-1* (NCBI Genbank X79688.1), *P. djamor a1-2* (NCBI Genbank AY462112.1), *P. djamor a2-2* (NCBI Genbank AY462112.1), *S. commune a_alpha_Y4* (EMBL-Bank M97181.1) and *S. commune a-alpha_Z4* (EMBL-Bank M97181.1). Additional searches were performed with MIP proteins of *P. djamor* (NCBI Genbank AY462112.1) and *P. nameko* (NCBI Genbank AB435542.1). Gene models for *FvHD1-1*, *FvHD2-1* and *FvHD2-2* were manually annotated. Predicted proteins for *FvHD1-1*, *FvHD2-1* and *FvHD2-2* were analyzed using COILS to identify dimerization motifs ([37] http://www.ch.embnet.org/software/COILS_form.html), WoLF PSORT to determine nuclear localization domains ([38] http://wolfpsort.org/) and 9aaTAD ([39], [40] http://www.es.embnet.org/Services/EMBnetAT/htdoc/9aatad/) to search for transactivation domains. NCBI Genbank accession numbers for the new *F. velutipes* genes are: *FvHD1-1*/*FvHD2-2* = HQ630588; *FvHD2-1*/*MIP* = HQ630589.

### Analysis of the Mat *A* locus

Tblastn searches were performed against a draft genome of *F. velutipes* (see above), the genome of *S. commune* strain H4-8, available at DOE Joint Genome Institute (http://genome.jgi-psf.org/Schco1/Schco1.home.html) and the genome of *Laccaria bicolor* S238N-H82 (available under the *Coprinus cinereus* project) using predicted proteins that surround the A mating-type locus of *C. cinerea* okayama 7#130, available under the *Coprinus cinereus* sequencing project of the BROAD institute of MIT (http://www.broadinstitute.org/annotation/genome/coprinus_cinereus/GenomesIndex.html). Gene homologues that were located on *F. velutipes* contigs Fv01174, Fv03236 and Fv02632 were used for comparison in Chromomapper 1.0.10.26 [41].

### Phylogenetic analyses

Pheromone receptor sequences of *L. bicolor* were obtained from the Joint Genome Institute (JGI) website according to Niculita-Hirzel *et al*. [31]. Sequences of *S. commune*, *P. djamor*, *C. cinerea* and *Cryptococcus neoformans* were obtained from NCBI Genbank using accession numbers as reported by James *et al*. (Figure 7, in [42]). Sequences of *F. velutipes* were obtained as described above. The protein sequences of pheromone receptors were aligned using ClustalW 1.64 [43] in combination with Gblocks 0.91b [44] to eliminate remaining poorly aligned sequences with a setting ‘allow smaller final blocks’. Ambiguously aligned regions were excluded from the protein sequences of pheromone receptors in the phylogenetic analyses. Maximum likelihood analyses were conducted using RAxML 7.04 [45]. A model of PROTGAMMAWAGF was selected with an analysis of ProtTest 2.4 [46] and incorporated in the analysis. Branch values from 1,000 nonparametric bootstrap repetitions were used for nodal support [47]. In this study, nodes were considered strongly supported when supported by more than 70% bootstrap value. The resulting tree was visualized with Figtree 1.3.1.

## Results

### Identification of pheromone receptor genes and pheromone precursors

**Figure 1:**
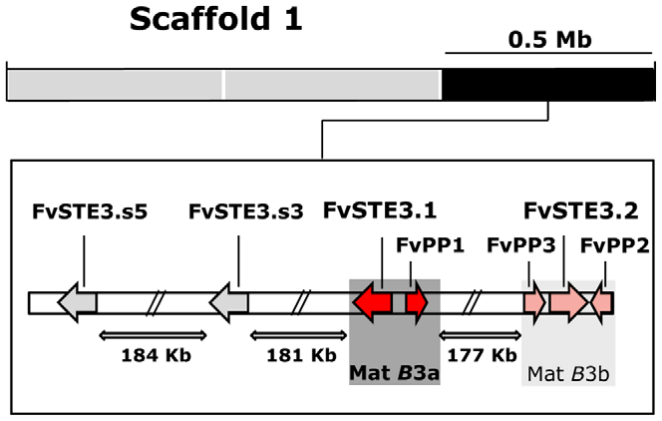
Organization of the mat*B*3 locus. The figure depicts a representative map of scaffold 1 with an enlarged section (black) for the mat*B*3 locus. Pheromone receptor genes are almost evenly spaced and cover one third of scaffold 1. *FvSTE3.1* and *FvPP1* specifically segregate with mat*B*3a (shaded dark grey) and are indicated by red arrows. Pheromone receptor *FvSTE3.2* and pheromones *FvPP2* and *FvPP3* (light red arrows) presumably form a second, mat*B*b sublocus (shaded light grey). Non mating-type specific pheromone receptors *FvSTE3.s3* and *FvSTE3.s5* (grey arrows) might be part of the mat*B* locus as well.

A draft genome of *Flammulina velutipes* monokaryotic strain KACC42780 (*A*3*B*3) was screened for pheromone receptor homologues and *CLA4,* a kinase gene shown to be linked to the *B* locus in other mushroom species [12]. Six contigs, five with a single and one with two pheromone receptor homologues were retrieved. *CLA4* was found on a separate contig (Table S1). Pheromone receptor genes were annotated and named *FvSTE3.1*, *FvSTE3.2* and *FvSTE3.s* one to five (*FvSTE3.s1* to *FvSTE3.s5*), where ‘s’ stands for ‘similar’ to distinguish non mating-type specific pheromone receptors (see below). Repeated searches with the newly indentified genes did not uncover additional pheromone receptors. Six predicted pheromone receptor proteins contained the seven-transmembrane domains that are characteristic for fungal pheromone receptors [26]. Alignments of the pheromone receptor proteins showed that the sequence of FvSte3.s2 deviated in the region that corresponded with the first transmembrane region in other pheromone receptors. Moreover, seven amino acids were deleted in this protein from a highly conserved motif, truncating its transmembrane sequence (not shown). FvSte3.s2 grouped specifically with FvSte3.s1 in maximum likelihood analyses (not shown), shared 60% base pair identity and was separated from gene *FvSTE3.s1* by only 6.7 Kb. All together, this suggests that *FvSTE3.s2* is a pseudogene that was derived as a copy from *FvSTE3.s1*. Three pheromone precursor genes named *FvPP1*, *FvPP2* and *FvPP3* were identified on contigs with pheromone receptors. *FvPP1* was located 2102bp apart from pheromone receptor *FvSTE3.1* and *FvPP2* and *FvPP3* flanked pheromone receptor *FvSTE3.2* at distances of 320bp and 427bp respectively (Figure 1). Notably, FvPp1 contained a C-terminal Tryptophan (W) behind its CAAX-box (CAAXW). All three FvPp proteins were classified as fungal pheromones according to Pfam 24.0 searches [36] with E-values of 0.0013, 0.45 and 0.035 for FvPp1, FvPp2 and FvPp3. Alignment of the FvPp proteins showed that FvPp2 (54 amino acids) and FvPp3 (46 amino acids) shared 55% and 54% reciprocal similarity in contrast to FvPp1 (54 amino acids) with 37%. The three proteins contained a glutamic acid/arginine (E/R) motif at amino acid position 13/14 (FvPp1) and 15/16 (FvPp2, FvPp3) counted from the C-terminus (Figure S1). These residues are conserved in various basidiomycetes and have been speculated to be the site of proteolytic cleavage (see introduction). Proteolytic splicing at the E/R sites, together with C-terminal processing, would result in peptides of nine (FvPp1) and 11 amino acids (FvPp2 and FvPp3) which corresponds well with the size of other fungal pheromones [19], [25].

### Segregation analysis of *FvSTE3* and *FvPP* genes

**Figure 2:**
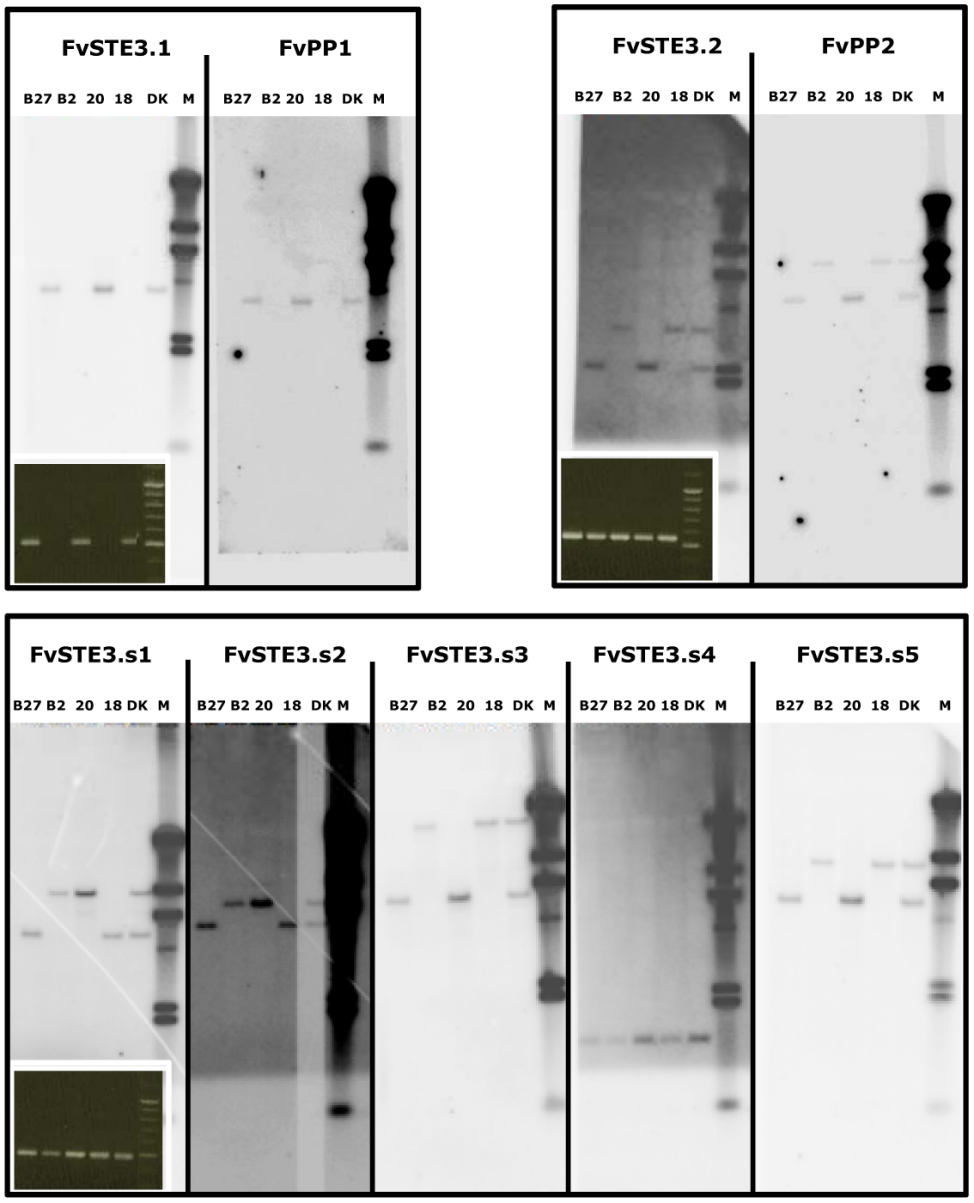
Distribution of pheromone receptors and pheromones in mat*B*3 and mat*B*4 loci. Southern blots confirmed the PCR distribution patterns of the pheromone receptors and pheromones in dikaryon KACC43778; *A*3*B*3*A*4*B*4 (DK), strain KACC43777; *A*4*B*4, (B18), KACC 42780; *A*3*B*3, (B20) and two monokaryotic siblings 4019-B2; *A*3*B*4, (B2) and 4019-B27; *A*4*B*3, (B27). Pheromone receptor *FvSTE3.1* and pheromone *FvPP1* are exclusively detected in strains containing the mat*B*3 locus (B20 and B27), and as a single copy in the dikaryon (DK). The small, equally sized signals for *FvSTE3.s4* on the Southern blot are caused by an internal SacI restriction site in this gene. PCR detection patterns for *FvSTE3.1*, *FvSTE3.2* and *FvSTE3.s1* are inserted as panels in the bottom of the Southern analyses. Strains that contained a copy of a gene invariably generated a specific PCR product. No false products were amplified with our primers. Pheromone receptor *FvSTE3.2*, *FvSTE3.s1* to *FvSTE3.s5* and pheromone receptors *FvPP2* and *FvPP3* were detected in mat*B*3 and mat*B*4 mating-types.

In order to designate the pheromone receptor and pheromone precursor genes to specific mat*B* loci, their distribution was analyzed in dikaryon KACC43778, parental strains KACC43777 (*A*4*B*4), KACC42780 (*A*3*B*3) and two monokaryotic siblings B2 (*A*3*B*4) and B27 (*A*4*B*3). PCR with specific primers (Table S2) detected *FvSTE3.1* and *FvPP1* explicitly in mat*B*3 strains. Pheromone receptors *FvSTE3.2*, *FvSTE3.s1* to *FvSTE3.s5* and pheromone precursors *FvPP2* and *FvPP3* were detected in mat*B*3 and mat*B*4 mating-types (Figure 2). Correct amplification and PCR patterns of *FvSTE3* and *FvPP* genes were confirmed by sequencing and Southern analysis (Figure 2). Analysis of 16 additional monokaryons with different mating-types showed that the observed distribution was persistent (Table 1). Monokaryons were derived as single spore colonies from dikaryon KACC43778 and mating-types were assigned based on clamp formation patterns (material and methods). Sequence alignment of the PCR products for each gene that were obtained from the 16 strains revealed two differing copies of genes *FvSTE3.s1*, *FvSTE3.s2* and *FvSTE3.s3*. This enabled segregation analysis through restriction fragment polymorphism of *FvSTE3.s1* and *FvSTE3.s2*. Subtype *FvSTE3.s1-a* was invariably detected together with *FvSTE3.s2-d* while *FvSTE3.s1-b* was linked with *FvSTE3.s2-c*. Segregation of the couples was independent from the analyzed mating-type loci (Table 1). Segregation of *FvSTE3.2*, *FvSTE3.s3* to *FvSTE3.s5*, *FvPP2* and *FvPP3* remained undetermined. Taken together, the results show that only a single pheromone receptor and pheromone segregate specifically with the *B*3 mating-type in *F. velutipes* KACC42780.

**Table 1:** Distribution of pheromone precursors, pheromone receptors and homeodomain genes in *F. velutipes* monokaryotic siblings.

Strain 4019-	DK	B10	B11	B13	B17	B19	B21	B22	B23	B25	B28	B30	B32	B34	B35	B36	B38
A mating-type	A3A4	A4	A4	A3	A3	A4	A4	A3	A4	A3	A3	A3	A4	A4	A3	A4	A4
B mating-type	B3B4	B4	B3	B3	B3	B3	B3	B4	B4	B3	B3	B4	B4	B3	B4	B4	B4
**Gene:**																	
FvSTE3.1	**+**	**−**	**+**	**+**	**+**	**+**	**+**	**−**	**−**	**+**	**+**	**−**	**−**	**+**	**−**	**−**	**−**
FvSTE3.2	**+**	**+**	**+**	**+**	**+**	**+**	**+**	**+**	**+**	**+**	**+**	**+**	**+**	**+**	**+**	**+**	**+**
FvSTE3.s1	**+**	**+**	**+**	**+**	**+**	**+**	**+**	**+**	**?**	**+**	**+**	**+**	**+**	**+**	**?**	**+**	**+**
RFLP s1-a/b		**b**	**a**	**a**	**a**	**b**	**b**	**a**	**?**	**a**	**a**	**b**	**a**	**a**	**?**	**a**	**a**
FvSTE3.s2	**+**	**+**	**+**	**+**	**+**	**+**	**+**	**+**	**+**	**+**	**+**	**+**	**+**	**+**	**+**	**+**	**+**
RFLP s2-c/d		**c**	**d**	**d**	**d**	**c**	**c**	**d**	**d**	**d**	**d**	**c**	**d**	**d**	**c**	**d**	**d**
FvSTE3.s2	**+**	**+**	**+**	**+**	**+**	**+**	**+**	**+**	**+**	**+**	**+**	**+**	**+**	**+**	**+**	**+**	**+**
FvSTE3.s3	**+**	**+**	**+**	**+**	**+**	**+**	**+**	**+**	**+**	**+**	**+**	**+**	**+**	**+**	**+**	**+**	**+**
FvSTE3.s4	**+**	**+**	**+**	**+**	**+**	**+**	**+**	**+**	**+**	**+**	**+**	**+**	**+**	**+**	**+**	**+**	**+**
FvSTE3.s5	**+**	**+**	**+**	**+**	**+**	**+**	**+**	**+**	**+**	**+**	**+**	**+**	**+**	**+**	**+**	**+**	**+**
Fvpp1	**+**	**−**	**+**	**+**	**+**	**+**	**+**	**−**	**−**	**+**	**+**	**−**	**−**	**+**	**−**	**−**	**−**
Fvpp2	**+**	**+**	**+**	**+**	**+**	**+**	**+**	**+**	**+**	**+**	**+**	**+**	**+**	**+**	**+**	**+**	**+**
Fvpp3	**+**	**+**	**+**	**+**	**+**	**+**	**+**	**+**	**+**	**+**	**+**	**+**	**+**	**+**	**+**	**+**	**+**
FvHD1-1	**+**	**+**	**+**	**+**	**+**	**+**	**+**	**+**	**+**	**+**	**+**	**+**	**+**	**+**	**+**	**+**	**+**
FvHD2-1	**+**	**−**	**−**	**+**	**+**	**−**	**−**	**+**	**−**	**+**	**+**	**+**	**−**	**−**	**+**	**−**	**−**
FvHD2-2	**+**	**−**	**−**	**+**	**+**	**−**	**−**	**+**	**−**	**+**	**+**	**+**	**−**	**−**	**+**	**−**	**−**

Distribution of pheromone receptors and pheromones was analyzed in monokaryotic F1 progeny from dikaryon KACC43778 (DK). Presence of genes is indicated by ‘+’ and absence by ‘-‘. *FvSTE3.s1* and *FvSTE3.s2* show the distribution of their respective, non digested PCR products. Their subtypes, genes that were identified in sequence analysis of *FvSTE3.s1* and *FvSTE3.s2* are *RFLPs1-a* (a), *RFLPs1-b* (b), *RFLPs2-c* (c) and *RFLPs2-d* (d). Subtypes were detected by restriction length polymorphism analysis of PCR products. The table shows specific linkage of pheromone receptor *FvSTE3.1* and pheromone *FvPP1* with the mat*B*3 locus. Copies of *FvSTE3.s1* and *FvSTE3.s2* recombine in pairs but independent from the mating-type loci; *FvSTE3.s1-a* is coupled to *FvSTE3.s2-d* and *FvSTE3.s1-b* is coupled to *FvSTE3.s2-c*. Homeodomain genes *FvHD2-1* and *FvHD2-2* co-segregate specifically with the mat*A*3 locus. Segregation of *FvSTE3.2*, *FvSTE3.s3*, *FvSTE3.s4, FvSTE3.s5, FvHD1-1*, *FvPP2* and *FvPP3* could not be determined. Copies of those genes were detected in all mating-types.

### Polymorphism of pheromone receptors

Mating-type specific genes in fungi are characterized by highly polymorphic alleles. We examined the distribution and polymorphism of the *FvSTE3* genes in various *F. velutipes* strains, from different countries (materials and methods). Genomic DNA, of two compatible monokaryons for each strain, was analyzed by PCR with specific primers (Table S2). *FvSTE3.s1*, *FvSTE3.s2*, *FvSTE3.s3* and *FvSTE3.s4* were frequently amplified with six, five, seven and seven products out of 10 strains. *FvSTE3.1*, *FvSTE3.2* and *FvSTE3.s5* were not detected outside the control (Table 2). New primer sets (Table S2, *additional sets) resulted in frequent amplification of *FvSTE3.s5* but *FvSTE3.1* and *FvSTE3.2* remained undetected. Sequences for the amplified genes shared respectively 92–99 percent base pair similarity (Table 2). Low polymorphism of *FvSTE3.s1* to *FvSTE3.s5* showed that those genes are not mating-type specific pheromone receptors; their amino acid sequences are identical. The absence of *FvSTE3.1* and *FvSTE3.2* from all tested *F. velutipes* strains, especially in comparison with the other pheromone receptors, clearly indicates polymorphic alleles for those two genes and therefore supports a mating-type specific role.

**Table 2:** Presence of pheromone receptors in different *F. velutipes* strains of wide geographical distribution.

Strain	Pheromone receptor						
	FvSTE3.1	FvSTE3.2	FvSTE3.s1	FvSTE3.s2	FvSTE3.s3	FvSTE3.s4	FvSTE3.s5
DK	**+**	**+**	**+**	**+**	**+**	**+**	**+**
4004 23	**−**	**−**	**−**	**+**	**+**	**+**	**+**
4004 32	**−**	**−**	**+**	**+**	**+**	**+**	**+**
4031 4	**−**	**−**	**+**	**+**	**−**	**−**	**+**
4031 10	**−**	**−**	**−**	**+**	**−**	**+**	**−**
4028 34	**−**	**−**	**+**	**+**	**+**	**+**	**+**
4028 38	**−**	**−**	**−**	**−**	**+**	**−**	**+**
4023 23	**−**	**−**	**+**	**−**	**+**	**+**	**−**
4023 29	**−**	**−**	**+**	**−**	**+**	**−**	**+**
4023 1	**−**	**−**	**−**	**−**	**+**	**+**	**+**
4023 5	**−**	**−**	**+**	**−**	**−**	**+**	**−**
Similarity	**−**	**−**	93–97%	92–98%	97–98%	93–99%	96–99%

The table shows a clear distinction in distribution between *FvSTE3.1*, *FvSTE3.2* and other pheromone receptors from strain KACC42780 when analyzed in geographically distant *F. velutipes* strains. Presence was determined by PCR analysis on genomic DNA of two compatible mating types of each strain. Locations of the strains were as follows: 4004−23 +4004−32, Korea; 4031−4+4031−10, Korea; 4028−34+4028−38, Taiwan; 4023−1+4023−5 & 4023−23+4023−29, *F. velutipes* var. *longispora*, USA. *FvSTE3.1* and *FvSTE3.2* are uniquely amplified from the control, dikaryon KACC43778. All other pheromone receptors are regularly amplified from geographically distant *F. velutipes* strains. Sequence similarity of PCR products for each regularly amplified pheromone receptor was high showing that they are non mating-type specific. Base pair similarity is shown in percentage in the bottom line.

### Phylogenetic analyses of pheromone receptors

**Figure 3:**
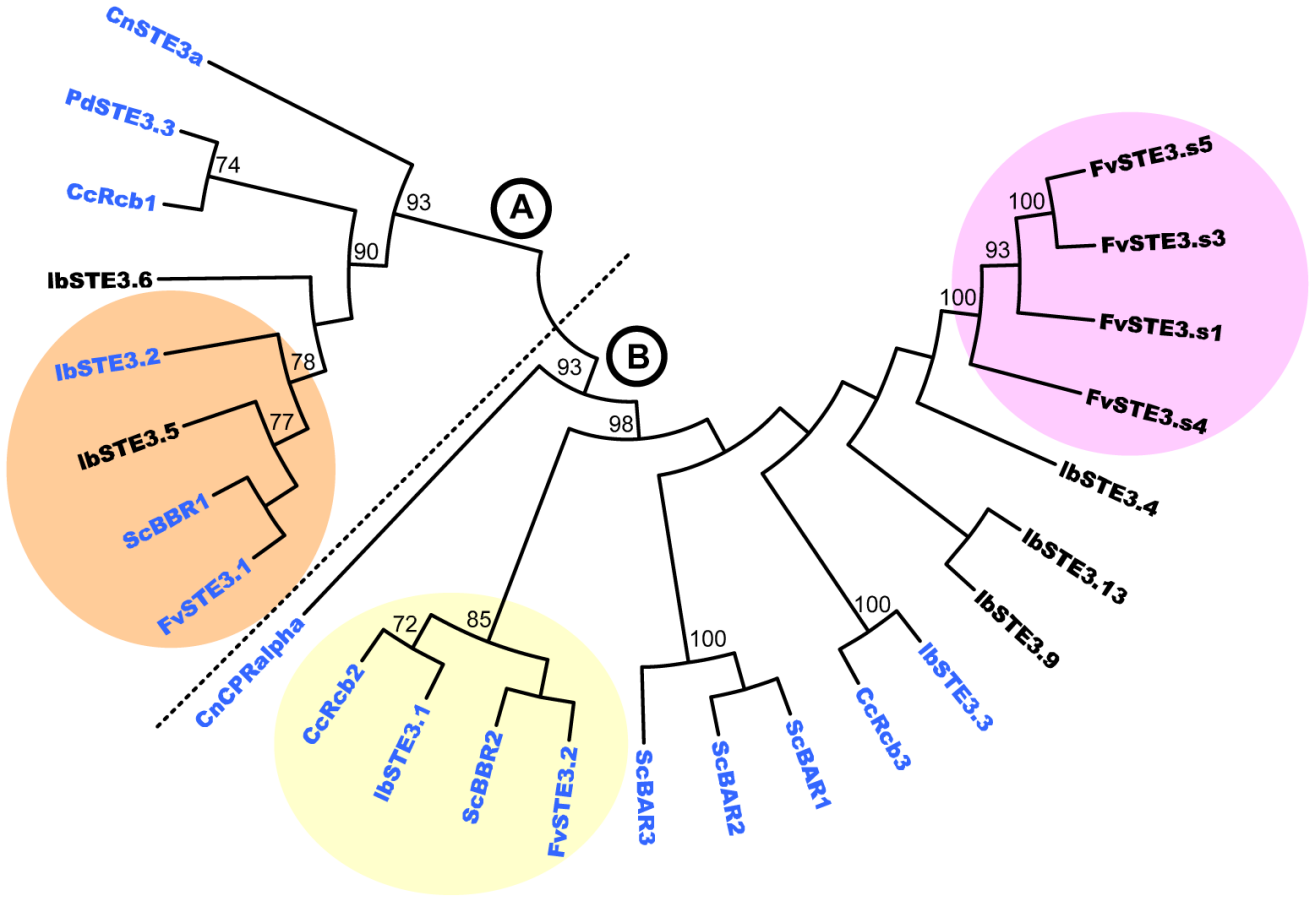
Phylogenetic tree of mating-type specific and non mating-type specific pheromone receptor protein sequences. The tree shows phylogeny amongst pheromone receptor proteins from *F. velutipes* (Fv), *C. cinerea* (Cc), *L. bicolor* (Lb), *S. commune* (Sc), *P. djamor* (Pd) and *C. neoformans* (Cn). Nodal supports with more than 70% bootstrap values are considered strongly supported and displayed in the tree. Known mating-type specific pheromone receptors are depicted in blue. Two major clades are distinguished, labeled A and B. The four non mating-type specific pheromone receptors of *F. velutipes* FvSte3.s1 to FvSte3.s5 (pseudogene *FvSTE3.s2* was excluded) form a separate group (shaded purple) within clade B that is supported by strong branch values. The clade including FvSte3.1 and two other known mating-type specific pheromone receptors is shaded in orange. The clade that contains FvSte3.2 is shaded in yellow. Both these clades are supported by strong branch values. FvSte3.1 and FvSte3.2 group closest with SCBbr1 and SCBbr2, respectively. Clades that contain known mating-type specific pheromone receptors are strong evidence for mating-type specificity of other clade members. LbSte3.5 is a notable exception.

Maximum likelihood analyses based on the protein sequences of *F. velutipes* pheromone receptors and that of other basidiomycete species indicated a division into two distinct clades (Figure 3, Clade A and B). The four pheromone receptors FvSte3.s1, FvSte3.s3, FvSte3.s4 and FvSte3.s5 formed a separate, strongly supported clade within clade B (Figure 3, purple shaded). FvSte3.2 was also grouped in clade B with strong support, together with pheromone receptor ScBbr2 from *S. commune*. In turn, they were strongly grouped with CcRcb2 and LbSte3.1 from *C. cinerea* and *L. bicolor* (Figure 3, yellow shaded). ScBbr2, CcRcb2 and LbSte3.1 are all mating-type specific pheromone receptors [18], [31]. FvSte3.1 was a close relative of ScBbr1 from *S. commune* in clade A and both were strongly grouped with LbSte3.2 and LbSte3.5 of *L. bicolor* which, except for LbSte3.5, are mating-type specific pheromone receptors as well. Close grouping of FvSte3.1 and FvSte3.2 with other known mating-type specific pheromone receptors formed a strong indication that they were also mating-type specific pheromone receptors. Interestingly, *ScBBR1* and *ScBBR2* are alleles at the same mat*B*β locus of *S. commune* [18], [23].

### Structure of the mat*B*3 locus

The pheromone receptor and pheromone precursor genes together with *CLA4* were mapped based on linkage of their respective contigs to a draft genome of *F. velutipes* KACC42780. The Mat*B*3*a* locus containing *FvSTE3.1* and *FvPP1* was located on scaffold 1 and flanked upstream by *FvSTE3.s3* and *FvSTE3.s5*, and downstream by *FvSTE3.2*, *FvPP2* and *FvPP3* (Figure 1). Notably, the pheromone receptors were almost evenly spaced by 177 Kb, 181 Kb and 184 Kb which was considerably more distant than has been reported for other basidiomycetes [31], [32]. Localization of the genes on the same fragment of scaffold 1 suggested that they might be part of the mat*B*3 locus. *FvSTE3.s1* and *FvSTE3.s2* were mapped on scaffold 26 and *FvSTE3.s4* on scaffold 29 (not shown). *CLA4* was located at 0.98 Mb and 1.16 Mb distance from the borders of scaffold 8 (not shown). This demonstrated that *FvSTE3.s1*, *FvSTE3.s2, FvSTE3.s4* and *CLA4* were not linked to the mat*B*3 locus.

### Identification of homeodomain genes

**Figure 4:**
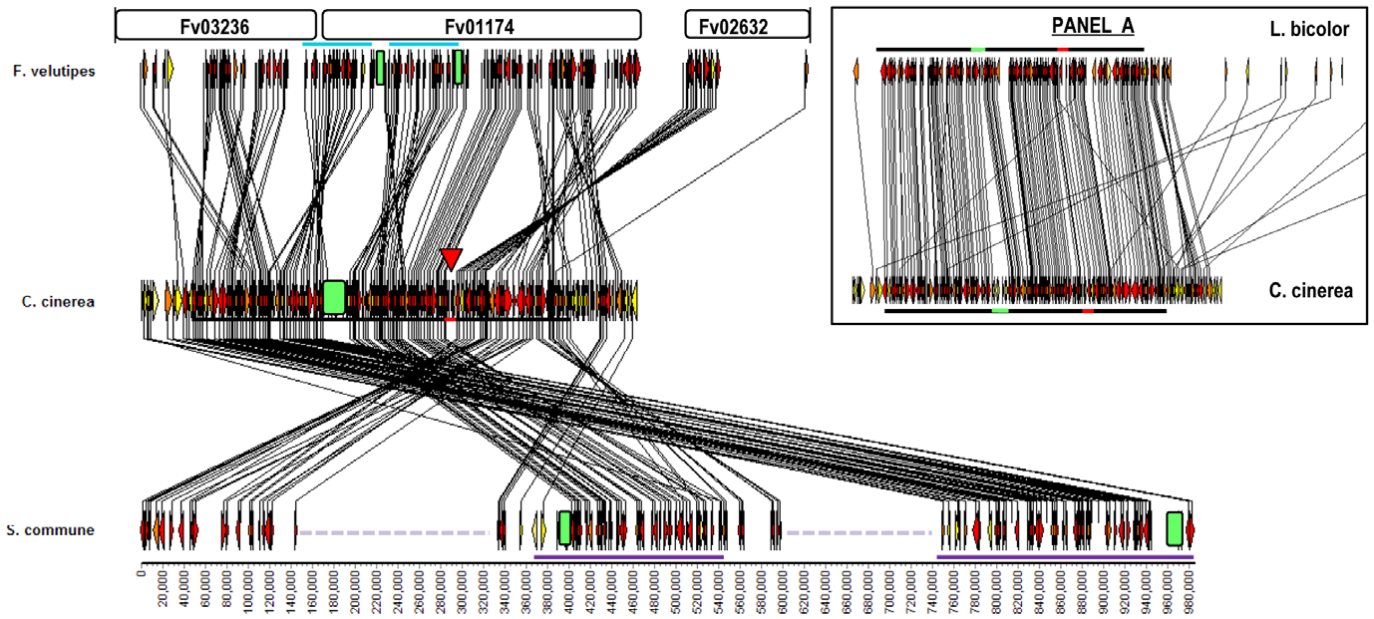
Synteny in mat*A* regions of *F. velutipes*, *C. cinerea*, *L. bicolor* and *S. commune*. Syntenic mapping of the mat*A* regions from *F. velutipes* (Fv), *C. cinerea* (Cc) and *S. commune* (Sc) in Chromomapper [41] reveals significant differences in gene arrangement. Distances are shown in kilo base pair (Kb) and *F. velutipes* contigs are depicted above the *F. velutipes* gene bar. A comparison of *C. cinerea* and *L. bicolor* (Lb) is shown in ‘panel A’ demonstrating the high synteny between those species. The black line under the gene bar of *C. cinerea* and above *L. bicolor* marks the 350 Kb, high syntenic region of in the figure and in panel A. Mat*A* loci of all species are indicated by green boxes. Rearranged regions in *F. velutipes* and *S. commune* are indicated by blue and purple lines respectively. Dashed light purple lines in *S. commune* indicate non syntenic regions. Borders of the highly syntenic region can be clearly recognized by yellow genes in *C. cinerea* and coincide both with borders of rearranged gene clusters in *F. velutipes* and *S. commune* and with individually rearranged genes in *L. bicolor* (panel A). Mat*A* loci (green) and a second spot (red triangle, red bars in panel A) are conserved sites of recombination in all four species. The gene order in *F. velutipes* and *S. commune* is changed by different events. *F velutipes* shows many inversions of gene clusters when compared to *C. cinerea. S. commune* shows rearrangements of larger, different gene groups. The overall gene orders of *F. velutipes* and *S. commune* are very similar to that of *C. cinerea* and *L. bicolor* which strongly suggests that these latter models represents an ancestral organization.

One *F. velutipes* HD1 gene (*FvHD1-1*), two HD2 genes (*FvHD2-1*, *FvHD2-2*) and the *Mitochondrial Intermediate Peptidase* (*MIP*) gene were identified in the *F. velutipes* genome located on a single contig; Fv01174 (Figure 4, Table S1). *MIP* was included because this gene is closely linked to HD genes in all Agaricomycetes [11], [12]. Analysis of the *FvHD2-1* and *FvHD2-2* gene models (accession codes in materials and methods) showed intron-exon distributions and long C-terminal exons similar to that of homeodomain gene *a2-1* and *b2-1* of *C. cinerea* [14]. The first and the second intron interrupted the homeodomain at the exact same locations in *F. velutipes* and *C. cinerea*. The second introns have been reported to be conserved in several other basidiomycetes as well [7], [14], [48], [49]. *FvHD1-1* (six predicted introns) showed no intron-exon resemblance to *C. cinerea* HD1 genes associated with *a2-1* and *b2-1*. No reliable coiled coils (less than 20–30% drops in the probability between weighted/non-weighted analysis) that could indicate dimerization motifs were found in the *F. velutipes* HD genes but nine-amino acid transactivation domains were detected in the N-termini of FvHd1-1, FvHd2-1 and FvHd2-2 (Table S3). These domains are generally found in mammalian and yeast transcription factors [40]. FvHd1-1, FvHd2-1 and FvHd2-2 were further predicted to contain single and or bipartite nuclear localization signals (Table S3).

### Segregation of *FvHD* genes

In order to link the FvHD genes to specific mat*A* loci we determined their distribution in mat*A*3 and mat*A*4 mating-types. Specific primers for *FvHD2-1*, *FvHD2-2* and *FvHD1-1* were designed for exon regions that flanked the conserved homeodomain (Table S2). *FvHD2-1* and *FvHD2-2* showed specific linkage to the mat*A*3 locus whereas *FvHD1-1* was detected both in strains with mat*A*3 and mat*A*4 loci (Table 1). PCR products (average 450bp) obtained from mat*A*3 and mat*A*4 strains with *FvHD1-1* specific primers, showed 97% base pair similarity and 100% amino acid identity (no polymorphism) demonstrating presence of a copy instead of different alleles. This means that the mat*A*3 locus lacks a specific HD1 gene in comparison with mat*A*4.

### Structure of the mat*A* locus

**Figure 5:**
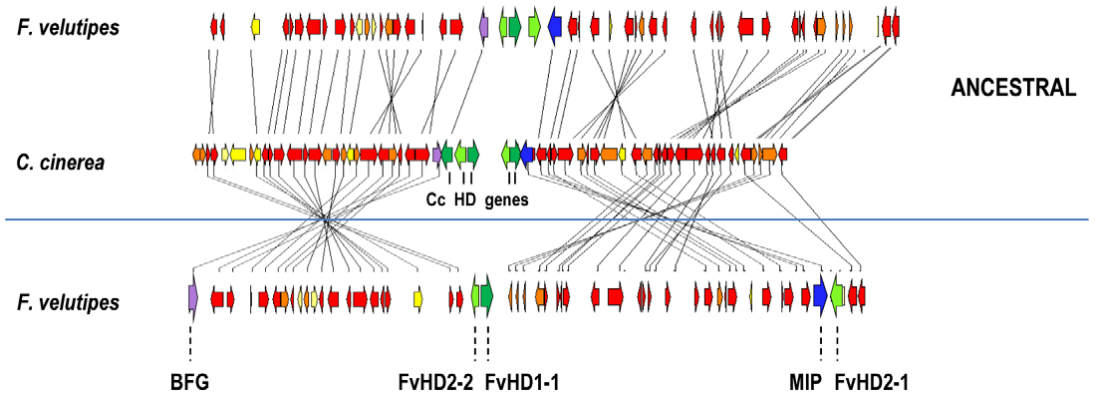
Gene order near the mat*A* locus of *F. velutipes* before and after inversion. A detailed overview of the synteny map in Figure 4, shows the individual genes of the mat*A* loci from *F. velutipes* and *C. cinerea*. Homeodomain gene *FvHD2-1* (green), the *Beta flanking gene* (BFG, purple) and the *Mitochondrial Intermediate Peptidase* (MIP, blue) are presently separated, flanking both sides of the *FvHD1-1/FvHD2-2* gene couple (bottom gene bar). The top gene bar shows the ancestral gene order in *F. velutipes*. Apart from a different number of HD genes, the matA locus is identical to that of *C. cinerea*. The synteny map clearly demonstrates that two inversions have caused separation of the mat*A* subloci in *F. velutipes*. Several additional inversions are indicated in the ancestral *F. velutipes* locus. Notably, *FvHD2-2* and *FvHD1-1* maintained their position during all changes.

*MIP* was located 201bp distant from *FvHD2-1* which is in contrast to most known basidiomycetes where *MIP* is directly flanked by a HD1 gene that is part of a HD1/HD2 couple [12]. Detailed screening of the sequence adjacent to *MIP* and *FvHD2-1* did not reveal a *FvHD1* gene but instead, a hypothetical gene with no orthologues near mat*A* subloci in *L. bicolor* (373Kb distant), *C. cinerea* (different chromosome) and *S. commune* (absent). Gene *FvHD1-1* and *FvHD2-2* that are 199bp apart, showed divergent, outward transcription directions (Figure 5) which is typical for HD1/HD2 couples in basidiomycetes [12]. *FvHD1-1* and *FvHD2-2* were separated by 73 Kb from *MIP* and *FvHD2-1*. Mat*A* subloci at large distance have so far only been reported for *S. commune* [4], [32]. No *Beta-flanking gene* was detected near the HD genes, which was unusual [12]. These deviations showed that the mat*A* locus had been subjected to several rearrangements relative to all other Agaricomycetes.

### Synteny of mat*A* regions

**Figure 6:**
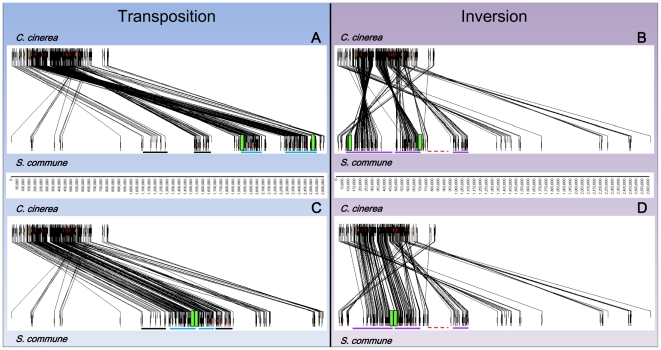
Synteny modeling of the events that separated the mat*A* subloci of *S. commune*. Panel A and B show synteny maps of *C. cinerea* and *S. commune*, with the latter in opposite orientations. Map A shows high gene order similarity whereas B suggests inversion of nearly all major *S. commune* segments. In map A, two large segments (blue lines) that correspond to the highly conserved 350 Kb mat*A* region (Figure 4) are displaced from the region flanked by the black lines. Repositioning of those fragments (in reversed order) reconstitutes the genomic organization also found in *C. cinerea* (C) with a single mat*A* locus (green) and a continuous high syntenic 350 Kb mat*A* region. Map B clearly shows two inversed gene clusters (purple lines) that have separated the *S. commune* mat*A* locus. Repositioning of those clusters (map D) reunites the mat*A* locus, but does not reunite the 350 Kb region. About one third (purple line most right, map D) of the genes corresponding to this region remains separated by a large non syntenic segment (∼250 Kb, red dotted line) and most other *S. commune* gene clusters still display inversed orientation in comparison to the gene order in *C. cinerea*.

To unravel the events that altered the organization of the mat*A* region of *F. velutipes* we mapped synteny of 200 successive genes surrounding the mat*A* locus of *C. cinerea* (chromosome 1, bp2471986-2934538) with that of *L. bicolor*, *F. velutipes* and *S. commune*. The latter was included because this fungus also has separated mat*A* subloci and is taxonomically closer to *F. velutipes* than *C. cinerea* and *L. bicolor* [50]. Since there had been no annotation of the *F. velutipes* genome, *C. cinerea* genes were acknowledged as “syntenic” in *F. velutipes* when similar protein sequences with expect values equal to or smaller than 10^−9^ were obtained by tblastn. Loss of syntenic genes in the mat*A* region from *C. cinerea* was indicated by 11 unique genes, and 21 (*L. bicolor*), 17 (*F. velutipes*) and 21 (*S. commune*) genes from *C. cinerea* that were not detected in one or two of these species. The selected *C. cinerea* region covered the mat*A* containing *F. velutipes* contig Fv01174 (304 Kb) as well as parts of contig Fv02632 and Fv03236, the last which was found to contain the missing *Beta flanking gene*. The three contigs were linked to scaffold 3 (3.8 Mb) of the *F. velutipes* draft genome where Fv01174 was flanked upstream at 2.1 Kb by Fv03236 and downstream by Fv02632 at 28 Kb (Figure 4). Our analysis identified synteny between *C. cinerea* and *L. bicolor* over a remarkably large segment of 350 Kb (Figure 4, panel A), much larger than previously demonstrated [31]. In addition, we identified the boundaries of this syntenic segment, which are clearly denoted by genes from *C. cinerea* that have no local homologue in *L. bicolor*, were differently distributed in *L. bicolor*, or were inverted (Figure 4, panel A). Those boundaries coincide with the ends of the syntenic part of the mat*A* region from *F. velutipes* (Figure 4). The mat*A* region in *F. velutipes* showed highest synteny over the first 250 Kb of the 350 Kb segment and somewhat lower in the last 100 Kb (Figure 4, black line, 250 Kb; start to red mark, 100 Kb; red mark to end). Though the specific gene order of *F. velutipes* in comparison to *C. cinerea* was changed by inversions, the overall gene order as found in *C. cinerea* was shown to be strongly preserved. None of the inverted gene groups was translocated (Figure 4). In *S. commune* the syntenic 350 Kb mat*A* region was also recovered, albeit in three different sections with 140 Kb and 200 Kb interval distances (Figure 4, purple and dashed light purple lines). Synteny was highest in the two segments corresponding to the first 250 Kb of the 350 Kb mat*A* region and lower for the third fragment, resembling *F. velutipes*. Moreover, synteny of the fragments in *S. commune* ended at the same relative locations that represented the synteny boundaries in *C. cinerea*, *L. bicolor* and *F. velutipes*. Finally, the gene order within the three segments was strongly conserved, indicating a high level of gene conservation in this entire region for all Agaricomycetes. Inside the 350 Kb segment, two spots showed high recombination in all four species. The first spot was the mat*A* locus itself, the second spot formed a small gap in synteny located between *C. cinerea* genes *CC1G_01875.3* and *CC1G_01877.3* (both hypothetical genes) and their respective orthologues. Exemplary, one of the few genes that was repositioned in *L. bicolor* in comparison to *C. cinerea* was inserted in this gap. In *F. velutipes* and *S. commune* gene clusters were separated by inversion or translocation at both of these locations (Figure 4, 5, 6). Detailed comparison of the *F. velutipes* gene order to that of *C. cinerea* showed that the mat*A* locus of *F. velutipes* was separated by inversion of two 70 Kb fragments directly left and right of *FvHD1-1* and *FvHD2-2* (Figure 4, 5). Modeling a reversion of those clusters reunited the *Beta-flanking gene*, *FvHD2-1*, *MIP, FvHD1-1* and *FvHD2-2* in a similar distribution as found in *C. cinerea* and *L. bicolor*. Notably, *FvHD2-2* and *FvHD1-1* were never moved during the rearrangements (Figure 5). Synteny mapping also revealed important differences between the mat*A* regions of *F. velutipes* and *S. commune*. First, all *S. commune* homeodomain genes were repositioned, whereas *FvHD2-2* and *FvHD1-1* retained their position during rearrangements. This shows that the respective mat*A* loci were split between different genes. Second, the *S. commune* fragments representing the first part of the high syntenic 350 Kb region, and whose rearangement caused separation of the mat*A* subloci, constituted 140 Kb and 260 Kb (Figure 4, purple lines). This was significantly larger than the 70 Kb in *F. velutipes* (Figure 4, blue lines) and shows that different fragments were rearranged. Third, the *S. commune* fragments were wedged by sections not syntenic to the 350 Kb *C. cinerea* mat*A* region (Figure 4, dotted light purple lines), which was not observed in *F. velutipes*.

To specify the events that split the *S. commune* mat*A* locus we extended the synteny map between *C. cinerea* and *S. commune* in both directions, using on average each next 5^th^ gene on the *C. cinerea* genome (Figure 6). Model A (Figure 6A) showed highest similarity to the gene order in *C. cinerea*, with all major *S. commune* gene clusters in an equal orientation. Model B (Figure 6B) with the *S. commune* chromosome in an opposite orientation, showed inversion of all but two major gene clusters. Both models indicated transposition of two (model A) or three (model B) high syntenic fragments corresponding to the 350 Kb region (Figure 6, blue and purple lines respectively). In addition, the two transposed fragments in model A were exchanged in order in comparison to *C. cinerea*. Repositioning of the two fragments representing the high syntenic 250 Kb region (discussed above) in model A, between the genes flanking the 350 Kb region and the genes representing the last 100 kb of the 350 Kb region on the other side (Figure 6A, C, black lines), reconstituted a mat*A* region mainly identical to *C. cinerea*. Model B clearly indicated inversions as a cause for separation of the *S. commune* mat*A* subloci. Indeed reversion of the genes underscored by the first (390 Kb) and second (210 Kb) purple line reunited the mat*A* subloci (Figure 6D). However, the *S. commune* mat*A* region that resulted from those inversions still contained a large non syntenic fragment (Figure 6, red dotted line) and differed considerably in gene order from *C. cinerea*.

## Discussion

With the fast growing range of sequenced genomes, new information of mating-type genes quickly becomes available. However, complete analyses of the genetic structure of mating-type loci in mushroom forming fungi remain rare. Our study focused on the genetic structure of the mating-type loci from *Flammulina velutipes* (Winter Mushroom or Enoki). This led to the finding of several characteristics for *F. velutipes* as well as new facts that will help gaining insight in mating-type locus evolution, especially in Agaricales.

### The *B* mating-type genes

Three pheromone precursors were identified near pheromone receptors in *F. velutipes*. As we limited our screen for pheromones to the six contigs that contained pheromone receptors (total 446 Kb), more pheromones can be expected to be found in the genome [31]. However, those pheromones will not be closely associated with the pheromone receptors and are unlikely to perform a mating-type specific role. FvPp1, the only pheromone precursor that was specifically linked to the mat*B*3 mating type is unique, ending with a tryptophan (W) after the CAAX-box. We suggest that this extra tryptophan originates from the former STOP codon (Stop = TAA, TAG or TGA). A single base pair substitution would suffice to change TAG or TGA to TGG (TGG = W). It is uncertain if the additional tryptophan impairs pheromone processing; yet deviant farnesylation signals have previously been demonstrated to be functional [23].

We identified two mating-type specific pheromone receptors (accompanying pheromones, phylogenetic grouping with other mating-type specific pheromone receptors and high sequence polymorphism) named *FvSTE3.1* and *FvSTE3.*2, which represented two of the three fungal pheromone receptors families. No genes of the third pheromone receptor family were detected in the mat*B*3 strain, though this family might be expected in other *F. velutipes* mat*B* loci.The identification of multiple non mating-type specific pheromone receptors in *F. velutipes* is in line with recent discoveries in *L. bicolor, C. cinerea* and *S. commune* [3], [31], [32]. We demonstrated a clear distinction between the two pheromone receptor types based on phylogenetic distribution and sequence polymorphism. Different sequence polymorphism indicates that these genes are subjected to different selection mechanisms. Somewhere in evolution, non mating-type specific pheromone receptors must have been functionally and selectively separated from mating-type specific ones. At the moment it is uncertain if non mating-type specific pheromone receptors are functional and what role they perform in the fungus. At least, their role is not mating-type specific (this study, [31], [32]).

### Structure of the mat*B* locus

Mating-type specific pheromone receptor *FvSTE3.2*, together with pheromone precursor *FvPP2* and *FvPP3*, was positioned in the same region of scaffold 1 as the mat*B*3a locus containing *FvSTE3.1* and *FvPP1* (Figure 3). Arguably, *FvSTE3.2* and the accompanying pheromone precursors comprise a functional second sublocus (yet our strain is just homozygous) since this specific receptor was not detected in any of the *F. velutipes* strains from different locations. The 177 Kb distance between both subloci makes the mat*B* locus of *F. velutipes* exceptionally large in comparison to other higher basidiomycetyes [20], [31], [32]. To this, non mating-type specific pheromone receptors have been demonstrated to be linked to mat*B* loci [3], [32] meaning that *FvSTE3.s3* and *FvSTE3.s5* could be part of mat*B*3 as well. This would increase the mat*B*3 locus to over 500 Kb. Identification of additional *F. velitupes* mat*B* loci should reveal if this large distance is consistent in *F. velutipes* and if *FvSTE3.s3* and *FvSTE3.s5* are truly connected.

The presence of *FvSTE3.1* and *FvSTE3.2* on different subloci corresponds with their phylogenetic separation into two clades that were derived through duplication [12]. It is surprising that their closest homologues, *ScBBR1* and *ScBBR2,* are alleles on the same locus (mat*B*β) in *S. commune* [18], [23]. The diversity between *F. velutipes* and *S. commune*, as well as the large distance in the first, shows that the genomic organization of mat*B* loci should be considered more flexible than previously has been assumed.

### The *A* mating-type genes

Three homeodomain genes distributed over two distant subloci were identified in *F. velutipes*. Both subloci were specifically linked to mating-type *A*3 (*FvHD2-1* and *FvHD2-2*) yet the only present HD1 gene (*FvHD1-1*) was found in mat*A*3 and mat*A*4 mating types. Mat*A*4 thus contains a different mat*A*a allele, consisting of *FvHD1-1* either combined with another HD2 gene than *FvHD2-2* or as a single gene. This means that *FvHD1-1* or *FvHD2-2*, that form a mating-type gene couple in mat*A*3, have been independently recombined in other mating types. Lack of a mat*A*3 specific HD1 gene dictates existence of at least one other HD1 gene in mat*A*4, presumably in the mat*A*b locus.

### Structure of the mat*A* region

We mapped the synteny between *F. velutipes*, *S. commune*, *L. bicolor* and *C. cinerea* based on blast searches with successive genes of the latter. Consequently, few genes that are missing in *C. cinerea* but that might be syntenic between other species remained undetected. The high detail of our maps however, showed that the applied method was accurate for both annotated and non annotated species. *F. velutipes* and *S. commune* showed different gene orders when compared, yet both followed the overall gene order of *C. cinerea* and *L. bicolor*. This, together with non separated mat*A* loci in *C. cinerea* and *L. bicolor* shows that they represent an ancestral organization. We identified a 350 Kb mat*A* region that is strongly conserved amongst Agaricales. Notably, synteny of genes belonging to this region is preserved even if parts become separated by chromosomal rearrangements as was shown in *S. commune*. The borders of the 350 Kb region, as well as two internal hotspots (one of which is the mat*A* locus) are conserved sites of recombination in two major clades of the Agaricales as classified by Matheny *et al*. [50]. They mark the edges of important rearranged segments in *F. velutipes* as well as *S. commune*. Reasonably, one might expect rearrangements of the strongly conserved 350 Kb mat*A* region in other Agaricales, especially in the Marasmioid clade that contains *F. velutipes* and *S. commune*.

Our segregation experiments showed that both mat*A*3 subloci are linked despite their 73 Kb distance. Until now, far distant mat*A* subloci in Agaricales were only known in *S. commune* (∼450 Kb) and considered to be an exception. The mat*A* subloci of *F. velutipes* were demonstrated to be separated by inversion of two (∼70 Kb) gene clusters and are clearly derived from an ancestral locus as represented by *C. cinerea*. It has been generally assumed that the mat*A* subloci of *S. commune* have followed a similar course leading to separation. However, our analysis showed significant differences between rearrangements in *F. velutipes* and *S. commune*. What is more, the analyses of the extended synteny map between *S. commune* and *C. cinerea* strongly indicate that the mat*A* subloci of *S. commune* were separated by transposition of gene clusters instead of inversions. As shown in model A (Figure 6A, C) a two step transposition of two high syntenic segments corresponding to the conserved 350 Kb mat*A* region, directly results in a gene order mostly similar to that in *C. cinerea*. Though it is possible to reconstitute the ancestral mat*A* locus by two inversions of considerably larger fragments as shown in model B, many rearrangements including transpositions remain (Figure 6B and D). Both the smaller sizes of the rearranged fragments and the fewer steps needed to reconstitute the ancestral gene order support the transposition model.

*F. velutipes* provides a phylogenetically diverse species with an unusual mating type system (one of two components of the mat*A*3 locus, HD2, is variable relative to mat*A*4 while the HD1 is identical and mat*B*3 contains a unique pheromone precursor). This enables comparative genomics to identify trends in mating type locus evolution. Studies of mating-type genes in Agaricales have shown that subloci are typically closely linked (10–20 kb) with the directly surrounding genes, especially in the mat*A* locus, being highly syntenic. In our study we determined that the Agaricales in fact have very large scale synteny at mat*A* (∼350 Kb) and that this synteny is maintained even when parts of this region are separated through chromosomal rearrangements (*S. commune*). Four conserved recombination hotspots allow reshuffling of large fragments of this region which resulted in separation of the mat*A* subloci of *F. velutipes* as well as of *S. commune*, by different events. This implies that separation of mat*A* loci is not exceptional and might be expected in other Agaricales. In addition to mat*A*, we determined that also mat*B* loci can exist over large distances (∼180 Kb) and that non mating-type specific pheromone receptors and mating-type specific ones are controlled by different selection mechanisms. Finally, the genes that were linked to specific mating types will serve as important molecular markers for breeding.

## Supporting Information

Figure S1 **Alignment of the three pheromone precursor proteins from** ***F. velutipes*** **KACC42780.** Conserved amino acids in the pheromone sequences are marked with *. The conserved putative proteolytic site represented by E and R is indicated by a bold line. The CAAX-box of each protein is designated by a dashed line. The C-terminal halves of FvPp2 and FvPp3 are highly similar (boxed).
(TIF)

Table S1 **Contigs of** ***F. velutipes*** **KACC42780 that were used in this study.** The table enlists the contigs of *F. velutipes* KACC42780 that were used in this study, describing their respective size and important genes that are located on those contigs. Genes that were specifically linked to mating type loci in this study are indicated with *. Pseudogene *FvSTE3.s2* is italicized. The STE3 prefix ‘Fv’ is added for *Flammulina velutipes*, the small ‘s’ preceding STE3 numbers is added to distinguish non mating-type specific pheromone receptors (FvSte3 *similar*) from mating-type specific pheromone receptors. Gene accession numbers for pheromone receptors, pheromones, homeodomain genes and *MIP* are given in the material and methods.
(DOC)

Table S2 **Primers used in this study.** List of specific primers for each pheromone receptor, pheromone and homeodomain gene from *F. velutipes* KACC42780. The first primer sets for each gene were used to determine distribution of genes for segregation analysis. The ‘*additional sets’ for *FvSTE3.1*, *FvSTE3.2* and *FvSTE3.s5* were newly designed after amplification in additional *F. velutipes* strains failed. Primer ‘Fl matA 1-1 rv 500bp’ for gene *FvHD1-1* is exceptional and anneals in an intron.
(DOC)

Table S3 **Amino acid positions of domains that were detected in proteins FvHd1-1, FvHd2-1 and FvHd2-2.** Homeodomain proteins were analyzed for 9 amino acid transactivation domains (9AA TAD) with 9aaTAD [40] and nuclear localization signals (NLS) by WoLF PSORT [38]. Numbers in the table refer to the amino acid (AA) numbers of the domains in the respective proteins.
(DOC)
