# Supplementary figures and images for: Comparative Genomics of the Mating-Type Loci of the Mushroom Flammulina velutipes Reveals Widespread Synteny and Recent Inversions
Source: PLoS One. 2011 Jul 20;6(7):e22249. doi: 10.1371/journal.pone.0022249 (PMC3140503; doi:10.1371/journal.pone.0022249)

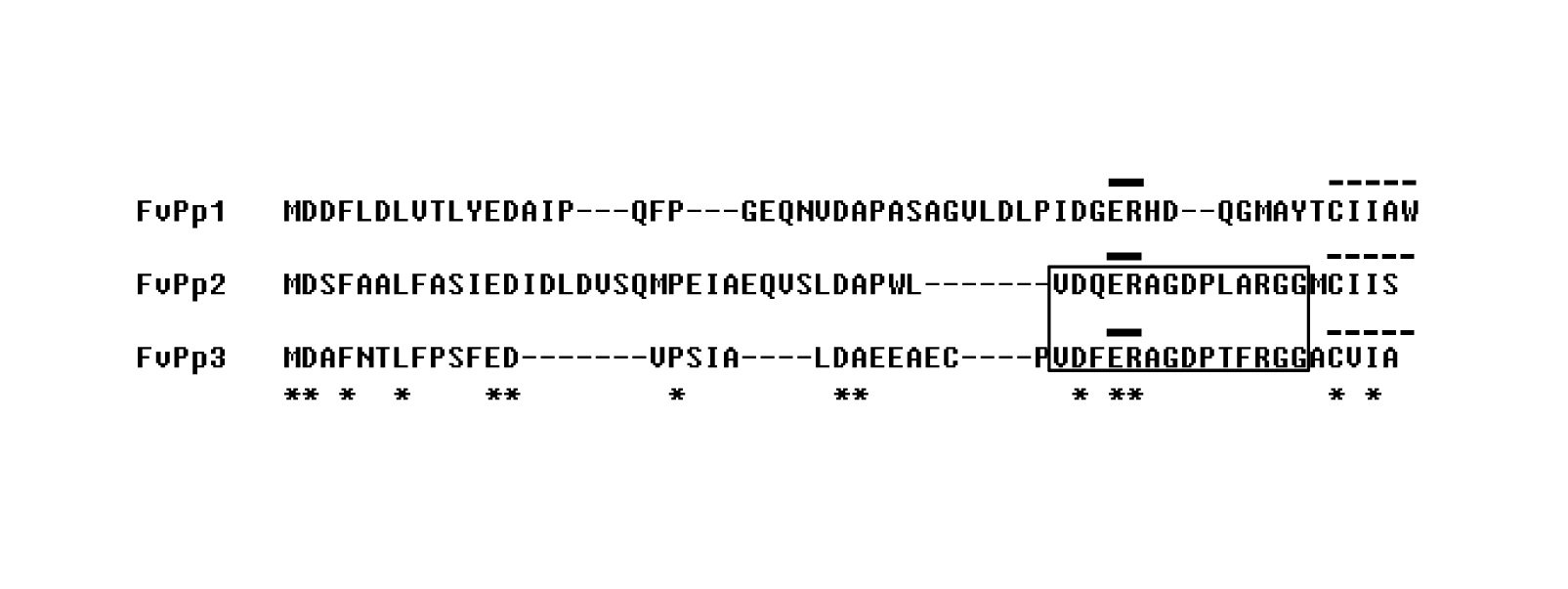

Supplement: Figure S1 — Alignment of the three pheromone precursor proteins from F. velutipes KACC42780. Conserved amino acids in the pheromone sequences are marked with *. The conserved putative proteolytic site represented by E and R is indicated by a bold line. The CAAX-box of each protein is designated by a dashed line. The C-terminal halves of FvPp2 and FvPp3 are highly similar (boxed). (TIF) [file pone.0022249.s001.tif]
