# Supplementary material for: Comparative Genomics of the Mating-Type Loci of the Mushroom Flammulina velutipes Reveals Widespread Synteny and Recent Inversions
Source: PLoS One. 2011 Jul 20;6(7):e22249. doi: 10.1371/journal.pone.0022249 (PMC3140503; doi:10.1371/journal.pone.0022249)
Supplement: Table S1 — Contigs of F. velutipes KACC42780 that were used in this study. The table enlists the contigs of F. velutipes KACC42780 that were used in this study, describing their respective size and important genes that are located on those contigs. Genes that were specifically linked to mating type loci in this study are indicated with *. Pseudogene FvSTE3.s2 is italicized. The STE3 prefix ‘Fv’ is added for Flammulina velutipes, the small ‘s’ preceding STE3 numbers is added to distinguish non mating-type specific pheromone receptors (FvSte3 similar) from mating-type specific pheromone receptors. Gene accession numbers for pheromone receptors, pheromones, homeodomain genes and MIP are given in the material and methods. (DOC) [file pone.0022249.s002.doc]

**Table S1. Contigs of *F. velutipes* KACC42780 that were used in this study.**

| ***F. velutipes* contig** | **Contig size** | **Gene** |
| --- | --- | --- |
| Fv01309 | 112 Kb | FvPP1 ***** |
|  |  | FvSTE3.1 ***** |
| Fv02445 | 19.2 Kb | FvSTE3.2 |
|  |  | FvPP2 |
|  |  | FvPP3 |
| Fv00493 | 120.3 Kb | FvSTE3.s1 |
|  |  | *FvSTE3.s2* |
| Fv01376 | 30.2 Kb | FvSTE3.s3 |
| Fv01839 | 137.1 Kb | FvSTE3.s4 |
| Fv03158 | 27.6 Kb | FvSTE3.s5 |
| Fv01174 | 304 Kb | FvHD1-1 |
|  |  | FvHD2-1* |
|  |  | FvHD2-2* |
|  |  | MIP |
| Fv01675 | 87 Kb | Cla4 |
| Fv03236 | 216.4 Kb | Beta flanking gene |
| Fv02632 | 168.6Kb | mat*A* region genes |
