# Supplementary material for: Comparative Genomics of the Mating-Type Loci of the Mushroom Flammulina velutipes Reveals Widespread Synteny and Recent Inversions
Source: PLoS One. 2011 Jul 20;6(7):e22249. doi: 10.1371/journal.pone.0022249 (PMC3140503; doi:10.1371/journal.pone.0022249)
Supplement: Table S2 — Primers used in this study. List of specific primers for each pheromone receptor, pheromone and homeodomain gene from F. velutipes KACC42780. The first primer sets for each gene were used to determine distribution of genes for segregation analysis. The ‘*additional sets’ for FvSTE3.1, FvSTE3.2 and FvSTE3.s5 were newly designed after amplification in additional F. velutipes strains failed. Primer ‘Fl matA 1-1 rv 500bp’ for gene FvHD1-1 is exceptional and anneals in an intron. (DOC) [file pone.0022249.s003.doc]

**Table S2. Primers used in this study.**

| **Gene** | **Primer name** | **Primer sequence** |
| --- | --- | --- |
| FvSTE3.1 | -matB -20 01309 Sth fw | 5' ACTACGTCGTACAAGGTCAC 3' |
|  | -matB -20 01309 Sth rv | 5' AGGCGAAGAATGCTACGAAG 3 |
| *additional set | -01309 polymorph scr Fw | 5' ACTACGAACTGCCCGTCGTC 3' |
|  | -01309 polymorph scr Rv | 5' CCATATCCGTCTGCGATGTC 3' |
| FvSTE3.2 | -matB -20 02445 Sth fw | 5' CACAGTTGTACACGCCCATC 3' |
|  | -matB -20 02445 Sth rv | 5' GTTTGTGGCCCACGAGTTTG 3' |
| *additional set | -02445 polymorph scr Fw | 5' CCCATCTTCGCGTTCTTAGG 3' |
|  | -02445 polymorph scr Rv | 5' CCGATATCTTCCAGGATGTC 3' |
| FvSTE3.s1 | -matB -20 00493A Sth fw | 5' TCGCAGACTTTACTGGATGG 3' |
|  | -matB -20 00493A Sth rv | 5' ACCCAAGGCCTATAGAAACG 3' |
| FvSTE3.s2 | -matB -20 00493B Sth fw | 5' TACGCGCATCATCCTGTCGG 3' |
|  | -matB -20 00493B Sth rv | 5' GGTGAAGAGCATCTCAGAAC 3' |
| FvSTE3.s3 | -matB -20 01376 Sth fw | 5' CATCCCGGATCCTTGTAGCG 3' |
|  | -matB -20 01376 Sth rv | 5' CGCGGCCATAATCATCAATC 3' |
| FvSTE3.s4 | -matB -20 01839 Sth fw | 5' TCTGTTTGGGCATCCCTCTC 3' |
|  | -matB -20 01839 Sth rv | 5' GGGAAGCGGGAATAAGATAG 3' |
| FvSTE3.s5 | -matB -20 03158 Sth fw | 5' GCATCCCGGATCATTATCGC 3' |
|  | -matB -20 03158 Sth rv | 5' AACAACGCAGACGACGTCAG 3' |
| *additional set | -03158 polymorph scr Fw | 5' CGAAGTATTCACGGCGTTTG 3' |
|  | -03158 polymorph scr Rv | 5' ATTGGGATTCCGAAGCAGAG 3' |
| FvPP1 | -Pprec 01309a probe fw | 5' TTCGTCGTAAATGGACGATTTC 3' |
|  | -Pprec 01309a probe rv | 5' AGCGCACAAACTTACCAAGC 3' |
| FvPP2 | -Pprec 02445a probe fw | 5' CGCCACTATGGACTCTTTCG 3' |
|  | -Pprec 02445a probe rv | 5' GCGGGCATTACGAGATGATG 3' |
| FvPP3 | -Pprec 02445b probe fw | 5' CAGCTATGGACGCATTTAAC 3' |
|  | -Pprec 02445b probe rv | 5' CGTCCTTCAGGCAATAACAC 3' |
| FvHD1-1 | -Fl matA 1-1 fw 500bp | 5' GAGGAGGTGATCCAAATAGC 3' |
|  | -Fl matA 1-1 rv 500bp | 5' TCTGCCGCACCTCTGTGTTG 3' |
| FvHD2-1 | -Fl matA 2-1 fw 500bp | 5' CTCTCAGCCTTCGCCATATC 3' |
|  | -Fl matA 2-1 rv 500bp | 5' TTTCGTCCTCCACGACTACC 3' |
| FvHD2-2 | -matA HD2 2 500bp Fw | 5' TTGTCGGGCCAATATCAAGG 3' |
|  | -matA HD2 2 500bp Rv | 5' TTCAGGCAGCGCTTAAAGTC 3' |
