# Supplementary material for: Comparative Genomics of the Mating-Type Loci of the Mushroom Flammulina velutipes Reveals Widespread Synteny and Recent Inversions
Source: PLoS One. 2011 Jul 20;6(7):e22249. doi: 10.1371/journal.pone.0022249 (PMC3140503; doi:10.1371/journal.pone.0022249)
Supplement: Table S3 — Amino acid positions of domains that were detected in proteins FvHd1-1, FvHd2-1 and FvHd2-2. Homeodomain proteins were analyzed for 9 amino acid transactivation domains (9AA TAD) with 9aaTAD [40] and nuclear localization signals (NLS) by WoLF PSORT [38]. Numbers in the table refer to the amino acid (AA) numbers of the domains in the respective proteins. (DOC) [file pone.0022249.s004.doc]

**Table S3. Amino acid positions of domains that were detected in proteins FvHd1-1, FvHd2-1 and FvHd2-2.**

|  |  | | |
| --- | --- | --- | --- |
|  | **FvHD1-1** | **FVHD2-1** | **FvHD2-2** |
| **Domain** |  |  |  |
| 9AA TAD | AA 56-64 | AA 131-139 | AA 12-20 |
| NLS single |  | AA 209-215 | AA 262-266 |
|  |  |  | AA 413-417 |
| NLS bipartite | AA 418-434 | AA 204-221 | AA 446-463 |
|  | AA 698-714 |  | AA 499-515 |
